# PERCEIVED NEIGHBORHOOD CHARACTERISTICS AND ACTIVITY ENGAGEMENT AMONG RACIALLY DIVERSE OLDER ADULTS

**DOI:** 10.1093/geroni/igae098.0779

**Published:** 2024-12-31

**Authors:** Jeein Jang, Jaqueline Avila, Jeffrey Burr

**Affiliations:** University of Massachusetts Boston, Boston, Massachusetts, United States; University of Massachusetts Boston, Boston, Massachusetts, United States; University of Massachusetts Boston, Boston, Massachusetts, United States

## Abstract

Engagement in social, cognitive, and physical activities is vital for healthy aging. Perceived neighborhood environment is associated with activity engagement, but there may be differences in this association between racial and ethnic groups. This study investigates the associations between neighborhood characteristics and activity engagement, emphasizing different activity domains and how race/ethnicity modifies this relationship. Using Health and Retirement Study data, the sample includes 9,180 adults aged 50 and above. The outcome is engagement in a list of 21 activities in the 2018-2020, measured as a total composite score and by domain (social, cognitive, and physical activities). The predictors are neighborhood social cohesion and physical disorder. Poisson regression analyses show a positive association between social cohesion and total activity engagement among older adults. Physical disorder is also positively associated with total activity engagement, suggesting that older adults in neighborhoods with high physical disorder may have tight-knit communities and strong bonds, working together to prevent further deterioration. Black older adults exhibit 1.03 times greater activity engagement than non-Hispanic Whites, while there were no differences compared to Hispanic individuals. Importantly, the association between social cohesion and overall activity engagement is weaker among Hispanics, even with the highest cohesion level. This suggests that Hispanics may prioritize individual autonomy in their activity choices over the influence of neighborhood social cohesion. Results by activity domains showed that the impact of social cohesion extended to each domain. Racial and ethnic differences warrant closer investigation in studies of neighborhoods and activity engagement in later life.

